# Growth, Survival and Reproduction of the Giant Clam *Tridacna maxima* (Röding 1798, Bivalvia) in Two Contrasting Lagoons in French Polynesia

**DOI:** 10.1371/journal.pone.0170565

**Published:** 2017-01-24

**Authors:** Simon Van Wynsberge, Serge Andréfouët, Nabila Gaertner-Mazouni, Colette C. C. Wabnitz, Mathilde Menoud, Gilles Le Moullac, Peva Levy, Antoine Gilbert, Georges Remoissenet

**Affiliations:** 1 Université de la Polynésie Française, UMR 241 EIO, Laboratoire d’Excellence CORAIL, Faa’a, Tahiti, French Polynesia; 2 Institut de Recherche pour le Développement, UMR 9220 ENTROPIE (Institut de Recherche Pour le Développement, Université de la Réunion, Centre National de la Recherche Scientifique), Laboratoire d’Excellence CORAIL, Nouméa, Nouvelle-Calédonie; 3 The Pacific Community (SPC), Noumea, New Caledonia; 4 Ifremer, UMR 241 EIO, Laboratoire d’Excellence CORAIL, Taravao, Tahiti, French Polynesia; 5 Ginger Soproner, Noumea, New Caledonia; 6 Direction des Ressources Marines et Minières, Fare Ute, Papeete, Tahiti, French Polynesia; James Cook University, AUSTRALIA

## Abstract

Shell growth, reproduction, and natural mortality of the giant clam *Tridacna maxima* were characterized over a two-year-period in the lagoon of the high island of Tubuai (Austral Archipelago) and in the semi-closed lagoon of Tatakoto (Tuamotu Archipelago) in French Polynesia. We also recorded temperature, water level, tidal slope, tidal range, and mean wave height in both lagoons. Lower lagoon aperture and exposure to oceanic swells at Tatakoto than at Tubuai was responsible for lower lagoon water renewal, as well as higher variability in temperature and water level at Tatakoto across the studied period. These different environmental conditions had an impact on giant clams. Firstly, spawning events in the lagoon of Tatakoto, detected by gonad maturity indices in June and July 2014, were timed with high oceanic water inflow and a decrease in lagoon water temperature. Secondly, temperature explained differences in shell growth rates between seasons and lagoons, generating different growth curves for the two sites. Thirdly, local mortality rates were also found to likely be related to water renewal patterns. In conclusion, our study suggests that reef aperture and lagoon water renewal rates play an integral role in giant clam life history, with significant differences in rates of shell growth, mortality and fertility found between open *versus* semi-closed atoll lagoons in coral reef ecosystems.

## Introduction

Among the 12 species of giant clams (Family Cardiidae, Subfamily Tridacninae), *Tridacna maxima* and *T*. *squamosa*, are widespread throughout the Indo-Pacific and can be found from the Red Sea and southeast Africa in the west to the Central Pacific in the east [1]. Depth range is also species-dependent, but individuals are usually restricted to shallow areas (< 7–10 m for *T*. *maxima*), where light provides them most of the energy necessary for survival, growth, and reproduction, owing to the ability of their symbiotic zooxanthellae to photosynthesize [2]. The geographical ranges of these two widespread species also cover a variety of reef types, including continental islands, open and closed atoll and submerged reefs, all characterized by very different biophysical environments [3].

Giant clams play various ecological roles in coral reef ecosystems [4]. Their tissues are food for a wide array of predators and scavengers, while their expelled zooxanthellae, faeces, and gametes are eaten by opportunistic feeders. The shells of giant clams provide substrate for epibionts, while commensal and ectoparasitic organisms live within their mantle cavities [4]. Finally, dense populations of giant clams produce large quantities of shell material which contribute to the complexity of the habitat structure. This is especially the case in semi-closed atolls of the Central Pacific that are characterized by high densities of *T*. *maxima* [5].

Most species of giant clams were traditionally exploited by local fishers for meat consumption or shell use as common household items [6]. Over the last decades, however, their commercial exploitation has expanded as demand for giant clam meat and shell has increased. To monitor international trade and contribute to the conservation of stocks, all Tridacninae were listed on Appendix II of the Convention on International Trade in Endangered Species of Wild Fauna and Flora (CITES) in the early eighties. Exports/imports of giant clams (alive, dead, tissue sample, etc.) between member countries are therefore strictly regulated.

The small giant clam *T*. *maxima* (max known size < 40 cm, but usually < 20 cm), is still found in relatively high densities at various locations throughout the Central Pacific [1]. *Tridacna maxima* is currently classified as lower risk/conservation dependent (i.e., species of ‘least concern’) under the IUCN Red List for threatened species. Densities and stocks can vary locally by several orders of magnitude, depending on past and present fishing pressure and fluctuations in environmental drivers [3]. The increasing commercial interest in *T*. *maxima*’s meat and the related depletion of their stocks throughout their range has highlighted the need for conservation measures designed to address the state of the resource at local levels. Thus, quantifying stocks and their spatial distribution are the first steps necessary to relevant management planning.

In the Central Pacific, *T*. *maxima* stock and individual density estimates of *T*. *maxima* were derived for a number of locations and reef types (e.g., Cook Islands, French Polynesia, Kiribati, Palau, Samoa; see [3] for a review). Specifically in French Polynesia, stock estimates for seven island and atoll lagoons were conducted *in situ* in 2004/2005 (Tubuai and Raivavae islands, Austral Archipelago; and Reao, Pukarua, Napuka, Tatakoto and Fangatau atolls, eastern Tuamotu [7]). Densities and size structures among lagoons were found to differ between reef types, with some atolls in the Tuamotu characterized by very dense aggregations of giant clams [8, 5]. At intra-lagoon scale *T*. *maxima* was found in a diversity of seascapes, either individually fixed on hard substrate or forming dense clusters of conspecifics on relatively loose substrate [5]. Based on findings, various habitat classes were described according to reef geomorphology, depth, and wave exposure, each characterised by its own density of giant clams [5, 9, 10]. The spatial characterization of stocks has led to the implementation of No Take Areas (NTA) (e.g., Tatakoto, [8]) and supported the development of aquaculture in atoll lagoons with high potential for spat collection (e.g., high densities, size structure oriented toward small individuals [7, 11]).

The spatial distribution of stocks, however, is only a snapshot of complex dynamics likely resulting from variations in life traits (i.e., growth, mortality, reproduction) and their interaction with the environment. When estimated at a specific point in time, stock distribution alone cannot provide an accurate view of population dynamics. Any conclusions drawn from such an assessment may therefore affect the success of long term management. For example, a re-evaluation of stocks at Tatakoto in 2012 highlighted a tenfold decrease in observed densities [12], with particularly extreme declines noted inside the NTA implemented in 2004. Results from the original assessments had highlighted this area as of priority interest for conservation, because of its record high clam densities and upstream location relative to the remainder of the lagoon, thus making it a potential source population for the entire lagoon [8]. However, unusual climatic conditions recorded in 2009 revealed that this area was also the most vulnerable to massive mortality events [12]. Several other mass mortality events have been reported from other atolls in the Tuamotu Archipelago, involving either dystrophic crises [13] or prolonged periods of high temperature (> 30°C during 5 months [14]). It has been argued that semi-closed lagoons of the Tuamotu Archipelago may be particularly sensitive to mass mortality events, as prolonged periods of low swell and low wind from the Southeast would prevent lagoon water renewal, normally maintained through water exchange along shallow channels located in the South part of the atoll rim [15]. Much less information is available from the literature for other Central Pacific countries, but a mass decline in *T*. *maxima* densities has also been reported for the enclosed lagoon of Millennium atoll (Kiribati; [16]). This remote and pristine atoll is not subject to strong fishing pressure, and the high variability of *T*. *maxima* densities is therefore assumed to be environmentally driven. To date, mass mortality events of giant clams driven by environmental changes have been mostly reported from closed to semi-closed atoll lagoons.

Besides the monitoring of stocks at regular time intervals, sustainable management also requires a clear understanding of population dynamics and biological processes, as well as their interaction with environmental factors at regional scale (e.g., climate), reef scale (e.g., closed or semi-closed lagoons versus open lagoons), and intra-lagoon scale (e.g., depth, wave exposure) [3]. However, to date, these are lacking for giant clams. In this study, we attempt to fill this gap by characterizing *T*. *maxima*’s life traits (shell growth, reproduction, and natural mortality) based on a sampling scheme spatially-structured at a number of scales. At lagoon and regional scale, our study sampled two sites in French Polynesia differing in their location and reef type. At intra-lagoon scale, the sampling scheme was designed according to the abiotic factors previously identified to structure the spatial distribution of densities at both locations [5]. We also took environmental measurements linked to local water renewal and discuss their impacts on *T*. *maxima* life traits at different scales.

## Methods

### Ethics statement

All *T*. *maxima* collections for this study were authorized by French Polynesia Ministerial Order no. 2850/MRM.

### Study sites

**Fig 1:**
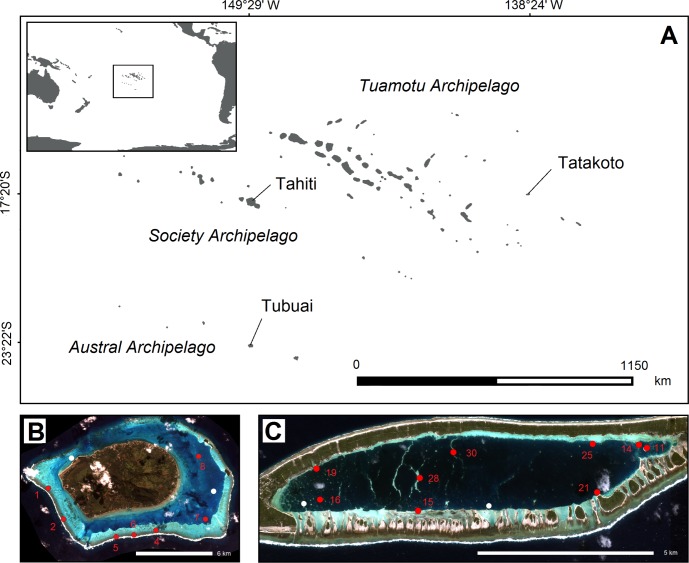
Location of studied sites. (A) Map of French Polynesia highlighting the location of Tatakoto in the Tuamotu Archipelago and Tubuai in the Austral Archipelago. (B-C) Satellite images and monitoring stations (red dots) at Tubuai and Tatakoto. For comparison, white dots indicate tagging stations used in a previous study [18].

Our study focused on two sites in French Polynesia: the semi-closed atoll of Tatakoto (138°24’W, 17°20’S) located in the Tuamotu Archipelago, 1,200 km East of Tahiti; and the island of Tubuai (149°29’W, 23°22’S) located in the Austral Archipelago, 600 km South of Tahiti (Fig 1A). Offshore surface temperatures range between 25–26°C in August and 27–28°C in February in the Eastern part of the Tuamotu Archipelago, and from 23–24°C (August) to 24–25°C (February) in the Austral Archipelago [17].

The two sites are also exposed to different wind regimes [17]: northeasterlies associated with high pressure systems and high temperatures are predominant almost year-round in the Tuamotu Archipelago, only typically extending to the Austral Archipelago from November to February (warm season). Strong westerlies associated with low pressure systems are the most common in the Austral Archipelago. The Austral islands’ cold season can also be associated with strong southeasterlies (40–50 km.h^-1^), bringing cool, dry weather that may last for several days. Such wind patterns are less common in the Tuamotu. These differences in wind patterns result in stark differences in annual rainfall and sunshine hours at both locations: around 2,000 mm.yr^-1^ and 2,200 h.yr^-1^ at Tubuai compared to 1,000 mm.yr^-1^ and 2,800 h.yr^-1^ at Tatakoto respectively [17, 19].

The two study sites are also representative of two contrasting reef aperture configurations (i.e., width of submerged area of the reef rim connecting the ocean and the lagoon [20]). The lagoon of Tubuai (90 km^2^) is open as it is permanently connected to oceanic waters through a large reef pass in the northern part of the lagoon, and a small reef pass at the southwest end of the lagoon (Fig 1B). All reef edges consist of submerged reef flats and ridges, therefore exposing the lagoon to waves at high tide and during periods of strong swell. To provide results representative of the lagoon, our study focused on seven stations spanning the western and southern reef flats (n = 2 and n = 2), the southern ridge (n = 1), and the eastern lagoon patch reefs (n = 2), all characterized by distinct giant clam densities [5] (referring to *T*. *maxima* in the context of this study). The atoll rim of Tatakoto is closed in its northern part, but the lagoon (11.46 km^2^) is connected to the ocean by way of several shallow channels that bissect the southern part of its rim (Fig 1C). Tatakoto is therefore a semi-closed atoll [20]. The highest densities of *T*. *maxima* were recorded in 2004 at the eastern extremity of the lagoon (up to 544 ind.m^-2^) leading to this area’s classification as a no take area (NTA) that same year [8]. Our study focused on nine stations at Tatakoto, each including a shallow area (< 1 m) and a medium-depth area (> 2–3 m), as depth was found to be the main determinant in structuring giant clam densities [5]. Two stations were located in the NTA (stations 11 and 14, Fig 1C).

### Characterisation of the lagoon environment

To characterize the physical environment of Tatakoto’s and Tubuai’s lagoons, we deployed temperature and pressure sensors at a number of locations at both sites. The exact positions, measurement types, and depths at which the sensors were deployed are summarized in Table 1. Calibration of all sensors by the same manufacturer allowed us to compare recorded values among instruments.

**Table 1:** Characteristics associated with the different temperature and pressor sensors deployed at Tatakoto and Tubuai. Station locations are provided in Fig 1B and 1C.

Station	Sensor n°	Sensor Type	Measurement	Depth (m)	Deployment time period
*Tatakoto*					
16	1	Seabird SBE56	Temperature	1.0	Nov2012-Oct2014
14	2	Seabird SBE56	Temperature	2.8	Nov2012-Oct2014
15	3	RBR RBRduo	Temperature & Pressure	3.4	Nov2012-Oct2014
11	4	RBR DR1050	Pressure	2.8	Jul2013-Oct2013
				2.8	May2014-Oct2014
*Tubuai*					
1	1	RBR DR1050	Pressure	1.2	Apr2013-Dec2014
8	2	RBR TGR1050	Pressure	1.3	Apr2013-Dec2014
7	3	RBR TWR2050	Temperature & Pressure	1.2	Apr2013-Dec2014
4	4	RBR TGR1050	Pressure	1.3	Apr2013-Dec2014
2	5	RBR TGR1050	Pressure	1.7	Apr2013-Dec2014

At Tatakoto, one sensor (RBRduo n°3) at station 15 recorded temperature every 30 minutes, and pressure in bursts of 512 measurements every 30 minutes from November 2012 to October 2014. Pressure measurements provided (i) a proxy for water levels inside the lagoon, (ii) tidal slope and tidal range, and (iii) mean wave height (H). Two further temperature sensors (SBE56 n°1 and n°2) and one pressure sensor (DR1050 n°4) were placed at opposite ends of the lagoon to characterize the wave exposure and thermal variations of these shallow areas. A greater sampling effort was allocated to temperature measurements at Tatakoto, as previous research has suggested this factor as critical to structuring the giant clam population in the atoll’s lagoon [12].

At Tubuai, one sensor (TWR2050 n°3) at station 7 recorded temperature every 30 minutes and pressure in bursts of 512 measurements every 30 minutes from April 2013 to December 2014. Four further pressure sensors (DR1050 n°1, TGR1050 n°2, n°4 and n°5) were also deployed at various locations to compare wave exposure between the western reef flat (sensors n°1 and n°5), the southern reef flat (sensor n°4), and patch reefs located in the eastern part of the lagoon (sensor n°2). The absolute values of the difference between two pressure measurements taken at one-minute interval (δP) were used as a proxy of wave exposure. Sampling at Tubuai focused on pressure (instead of temperature) as wave exposure has previously been identified as the main factor structuring *T*. *maxima* densities in this lagoon [5].

### Shell growth and natural mortality

We estimated growth and natural mortality of giant clams through regular monitoring of tagged individuals.

At Tatakoto, where individuals occur in high density aggregations, we used a tagging technique commonly referred to in the literature [21, 22, 23, 24, 25, 26, 18]: a plastic tag affixed to the external face of the shell with epoxy glue. The method required animals to be out of the water for between 15 to 30 minutes. Giant clams that showed any sign of stress (bleaching of the mantle) one week after tagging were systematically excluded from the analysis. A complementary study specifically designed to test for the effect of tagging on natural mortality is available as Supporting Information (S1 Appendix). Overall, 238 giant clams were tagged in November 2012. Their survival was checked, and maximum shell length (anterior-posterior measurements) estimated to the nearest millimetre with callipers in July 2013, October 2013, May 2014, and October 2014. Supplementary tagging (n = 98 individuals) was undertaken in July 2013 since a high proportion of initially tagged individuals were found dead or could not be relocated during subsequent monitoring campaigns (cf. Results and S1 Appendix for a discussion).

At Tubuai, the lower densities of giant clams allowed us to implement a method that is not stressful for the animals. Individuals were identified by their colours and their position along permanently established transects. Overall, 424 individuals were virtually tagged in April 2013. We checked whether individuals were still alive and estimated their shell length (anterior-posterior measurements) to the nearest millimetre using callipers in November 2013, May 2014, and December 2014. No supplementary tagging was needed at Tubuai as the majority of initially tagged clams were relocated in resurveys.

Analyses of natural mortality and shell growth were first performed at lagoon-scale (i.e., all stations confounded) for the entire study period. For considerations at intra-lagoon-scale, we structured the analyses by the same factors that structured sampling (station, depth, and season). The monitoring field visits allowed us to define four time periods at Tatakoto and three at Tubuai, each including either the coldest time of the year (July-September, hereafter termed “cold season”) or the warmest (December-March, hereafter termed “warm season”).

Shell growth rates (*G*) between field visits were calculated using the following equation:

$$G=\frac{L_{t+\Delta t}-L_{t}}{\Delta t}$$ (eq. 1)

where *L*_*t*_ is the shell length (anterior-posterior measurement, in cm) at time t and *Δt* denotes the time interval between two measurements (in days). The instantaneous natural mortality rate (*M*) was calculated using Eq 2:

$$N_{t+1}=N_{t}\times e^{-M(\Delta t)}$$ (eq. 2)

where *N*_*t*_ and *N*_*t+1*_ are the number of survivors between the start and the end of the time period considered. We assumed for giant clams that “disappeared” between two sampling events to be dead in our analyses (see Supporting Information S1 Appendix for a discussion). As fishermen were observed harvesting clams from Station 30 at Tatakoto (see Fig 1C for the location of station 30) between October 2013 and May 2014, and between May 2014 and October 2014, station 30 was removed from all analyses. As no other observations indicative of clam harvesting at tagging stations were noted or reported, all marked clams considered in the analyses were assumed not subject to fishing pressure during the study period.

### Reproduction

At Tatakoto, we evaluated the temporal variation in reproductive activity based on the gonad status of 15 specimens collected monthly from August 2013 to July 2014 (i.e., 11 months), from the western part of the lagoon. The sampling location was chosen for practical and logistical reasons. At Tubuai, the monitoring of gonad maturation could not be conducted in as systematic a fashion as at Tatakoto because of operational constraints. Thus, samples were collected opportunistically 5 times over a two year period: August 2013, November 2013, September 2014, December 2014, and August 2015.

Specimens were measured, weighed, and dissected to determine shell length, the wet weight of flesh *W*_*biomass*_ (i.e., after the byssus had been removed), and the gonad weight *W*_*gonad*_, to calculate the “Gonado-Somatic Index” (GSI) (Eq 3).

$$GSI=\frac{W_{gonad}}{W_{biomass}}$$ (eq. 3)

The proportion of female *versus* male tissues (The “Gonadal Sex Ratio”, GSR) and the proportion of male tissues harbouring spermatozoids (SPZ) were also calculated using histological treatments and used as proxy of sexual maturity, following the procedure recommended by a recent study [27].

### Modelling and statistical analyses

We calculated the mean temperature over the study period ($\bar{T}$) and the intensity of a seasonal signal (*T*_*v*_) from the recorded values of temperature (*T*) using the following equation:

$$T=\bar{T}+T_{v}\times \sin\left(\frac{2\pi }{365}\times (t+a)\right)$$ (eq. 4)

The mean (±SE) for parameters $\bar{T}$ and *T*_*v*_ were derived using the nls function of the stats package in R.3.1.0, and used to test for differences in mean temperature and seasonal variation between Tatakoto and Tubuai. We compared the short-term variation in temperature between the two sites by using the variance of residuals. These analyses were only performed for time series common to both locations.

At the scale of the lagoon, shell growth rate (*G*) and instantaneous rate of natural mortality (*M*)—both calculated over the entire study period—were expressed as a linear model of mean shell length (function lm of stats package in R.3.1.0). To allow for comparison of shell growth rates published elsewhere, the estimates of intercept and slope were used to calculate the growth parameter *k* and the asymptotic length at which growth is zero (*L*_∞_) of the Von Bertalanffy growth equation:

$$L_{t}=L_{\infty }(1-e^{-k(t-t_{0})})$$ (eq. 5)

At intra-lagoon scale, instantaneous mortality rates (*M*) were square-root transformed and expressed as a function of seasons, stations, depth and interactions between these factors on the basis of a linear model. Shell growth rates (*G*) calculated over each monitoring period were expressed as a function of shell length and sampling-structuring factors (stations and season for Tubuai; station, season and depth for Tatakoto) using a linear mixed model (function lme of nlme package in R. 3.1.0.). We could not test for interactions between factors, but a random effect considered the existence of dissimilarities between individuals (i.e., individual specific effect). For all models, we checked the assumption of homogeneity of variance and normality of residuals graphically using normal quantile-quantile plots and scale-location plots of sqrt(|residuals|). The significance of each parameter was tested using an analysis of variance (anova function in R.3.1.0.).

## Results

### Characterisation of the lagoon environment

**Fig 2:**
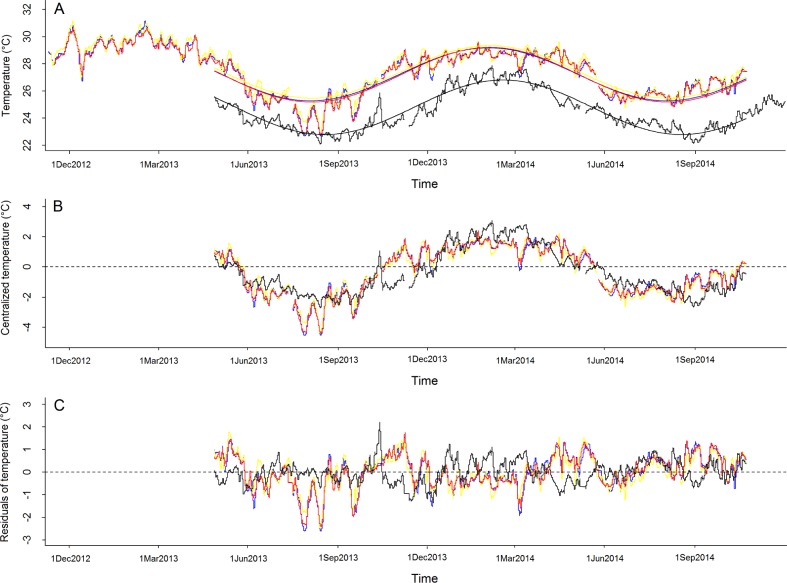
Temperature series recorded at Tatakoto and Tubuai over the timeframe of this study. (A) Raw data; (B) Data centralized to their mean; (C) Data centralized to their mean and seasonality removed (i.e., residuals of models as estimated using Eq 4). Grey lines: sensor n°1 (Tatakoto); Yellow lines: sensor n°2 (Tatakoto); Red lines: sensor n°3 (Tatakoto); Black lines: sensor n°3 (Tubuai). See Table 1 and Fig 1 for location and depth of sensors. Predicted values of models defined by Eq 4 appear as smooth lines in the upper panel.

At lagoon-scale, temperatures were systematically higher (2.6°C on average) at Tatakoto than Tubuai (Fig 2A). Seasonal variability was similar between the two sites (Fig 2B), with mean (± SE) values for (*T*_*v*_) estimated at 2.02 ± 0.007 for Tatakoto and 2.01 ± 0.004 for Tubuai. Even though the temperature sensor n°3 at Tatakoto was located slightly deeper than the temperature sensor at Tubuai (Table 1), the short term variation in temperatures was higher for sensor n°3 at Tatakoto than at Tubuai (Fig 2C); with a residuals’ standard deviation of 0.75 and 0.58 respectively.

**Fig 3:**
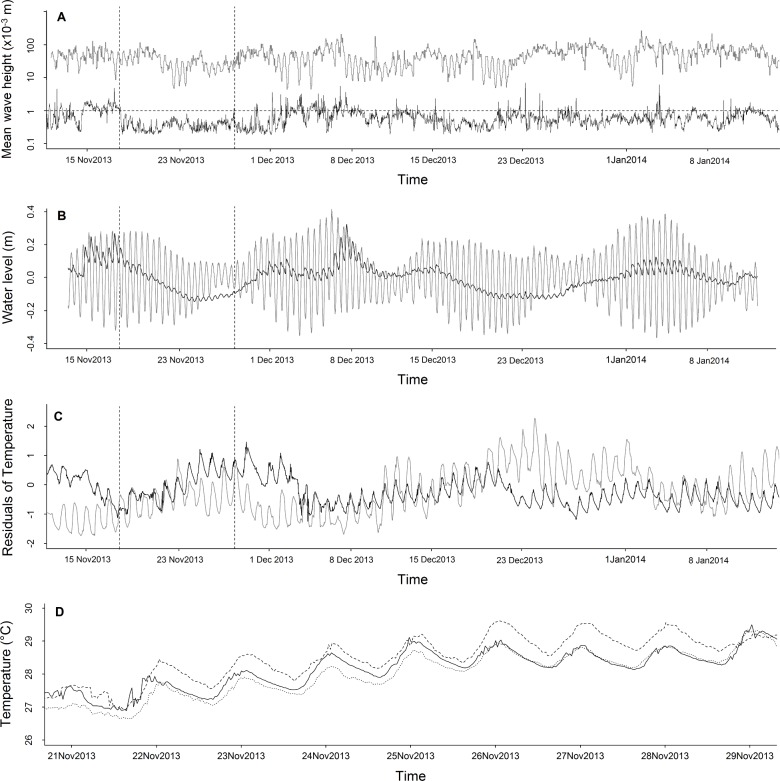
Physical characteristics of Tatakoto and Tubuai lagoons during the study period. (A) Mean wave height, (B) water level (C) residuals of temperature (mean and seasonality removed) recorded by sensors n°3 (see Table 1 and Fig 1) at Tatakoto (black) and Tubuai (grey) during the 2013–2014 warm season. (D) Temperature series for the three sensors deployed at Tatakoto during a 10-day period of low wave height (< 10^−3^ m; indicated by dashed lines) (dashed line: sensor n°1, spaced dashed line: sensor n°2, solid line: sensor n°3).

The tidal slope was an order of magnitude higher at Tubuai than Tatakoto, with 5% and 95% quantile values at -0.12 m.h^-1^ and 0.13 m.h^-1^ respectively for Tubuai compared to -0.03 m.h^-1^ and 0.04 m.h^-1^ for Tatakoto. Analyses highlighted that a 10-day period of low mean wave height (< 1×10^−3^ m) at Tatakoto (Fig 3A) was associated with an important decline in lagoon water levels (from +0.112 to -0.041 m relative to the mean water level, Fig 3B), a decrease of tidal slopes toward zero inside the lagoon (tidal range < 0,05 m; see the bi-daily oscillation on Fig 3B), and a marked increase in water temperature (from 26.6°C to 29.3°C, Fig 3D). At Tubuai, the duration and strength of low mean wave height periods also influenced temperature, but to a lesser extent. Low wave periods were uncommon and only of short duration (mean wave height always > 1×10^−3^ m), and the water level of the lagoon was permanently influenced by tide (tidal range consistently over 0.2 m).

At intra-lagoon-scale, the three temperature sensors deployed at Tatakoto yielded similar results despite being placed at different locations and depths (ranging from 1 to 3.4 m, Table 1; Fig 2). Temperatures recorded at mid-day by the sensor located in the eastern portion of the basin were higher compared to the other sensors (mean ± SD of differences between sensor n°2 and sensor n°1 over the entire time series: 0.32 ± 0.37°C, Fig 3D). At Tubuai, wave exposure varied markedly depending on location, with the highest variability in pressure recorded on western reef flats (δP = 0.041 ± 0.041 (SD) for sensor n°5 and δP = 0.027 ± 0.026 for sensor n°1) and southern reef flats (δP = 0.025 ± 0.027 for sensor n°4). Patch reefs located in the southeast part of the lagoon were more sheltered, with δP values ranging from 0.0096 ± 0.0063 for sensor n°3 and 0.021 ± 0.019 for sensor n°2.

### Shell growth

Results for shell growth rates (*G*) showed high variability. However, on average, growth rates (± SD) at lagoon-scale were higher and more variable during the warm season (1.84×10^−3^ ± 3.21×10^−3^ cm.d^-1^ and 2.29×10^−3^ ± 2.68×10^−3^ cm.d^-1^ for Tatakoto and Tubuai respectively) than the cold season (0.83×10^−3^ ± 2.20×10^−3^ cm.d^-1^ and 1.94×10^−3^ ± 2.15×10^−3^ cm.d^-1^ for Tatakoto and Tubuai respectively). The seasonal difference in mean growth was more pronounced at Tatakoto, but remained significant at both sites (p < 0.001 and p < 0.01 for Tatakoto and Tubuai respectively).

**Fig 4:**
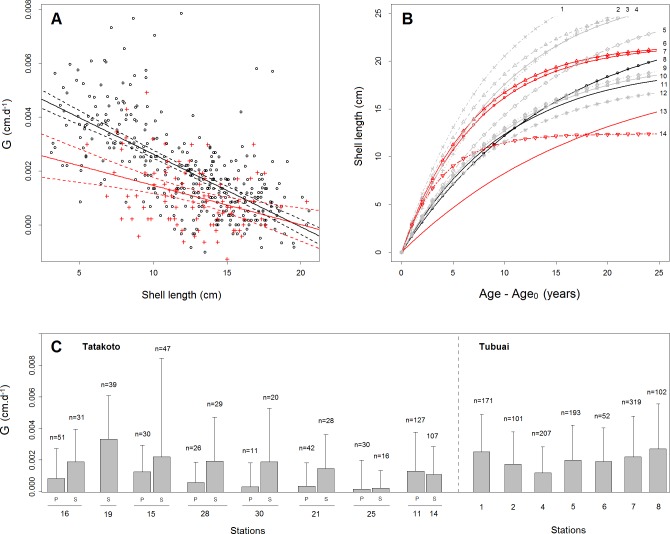
Growth rate and growth curve of giant clams for the two studied sites. (A) Predicted means (solid lines) and confidence intervals (dashed lines) for growth rate (*G*) as a function of mean shell length for *Tridacna maxima* at Tatakoto (red, n = 93) and Tubuai (black, n = 352). Shell growth rate G (cm.d^-1^) is the ratio between shell length increment and number of days between two measurements (see methods). (B) Growth curves obtained from data collected in this study (curve n°13 for Tatakoto and curve n°11 for Tubuai) and information gleaned from the literature. 1: Papua New-Guinea [28]; 2: Tonga [23]; 3: Solitary islands [29]; 4: Rose atoll [30]; 5: One Tree Island [21]; 6: Fangatau [18]; 7: Tatakoto [18]; 8: Tubuai [18]; 9: Aitutaki [25]; 10: Manihiki [25]; 12: Suwarrow [25]; 14: Takapoto [31]. (C) G (mean ± SD) estimated from sampling at each station. Stations are further subdivided into a deep site (P) and a shallow site (S) at Tatakoto.

Shell growth rate was found to decline significantly with increasing shell length (p < 0.001 at both sites, Fig 4A), but the relationship exhibited high variability (R^2^ = 0.44 for Tubuai and R^2^ = 0.13 for Tatakoto). Means (± SE) for the Von Bertalanffy parameter k was higher at Tubuai (2.7×10^−4^ ± 1.6×10^−5^ d^-1^) than Tatakoto (1.5×10^−4^ ± 3.9×10^−5^ d^-1^), yielding contrasting growth curves (Fig 4B). However, estimates of *L*_∞_ were comparable at both sites (19.7 and 20.0 cm for Tubuai and Tatakoto respectively).

At intra-lagoon-scale, growth rates were significantly different between stations (p < 0.001 for both sites, Table 2), with lower values recorded for station 25 at Tatakoto and for station 4 at Tubuai (Fig 4C). At Tatakoto, the shell growth rate was systematically higher for shallow sites (< 1 m) compared to deeper sites (p < 0.01), except for the two easternmost stations (stations 11 and 14) where the entire basin is relatively shallow (< 5 m).

**Table 2:** ANOVA results for the linear mixed model (lme) testing for differences in shell growth (*G*) among shell lengths and factors structuring sampling.

Site	Factor	Df	F	p-value
Tatakoto	Station	8	04.26	<0.001***
	Depth	1	10.37	<0.01**
	Season	1	21.69	< 0.001***
	Shell length	1	37.71	< 0.001***
Tubuai	Station	6	05.80	<0.001***
	Season	1	06.82	<0.01**
	Shell length	1	330.12	< 0.001***

* Denotes a significant difference with a type I (α) error below * 0.05, ** 0.01 and *** 0.001.

### Reproduction

**Fig 5:**
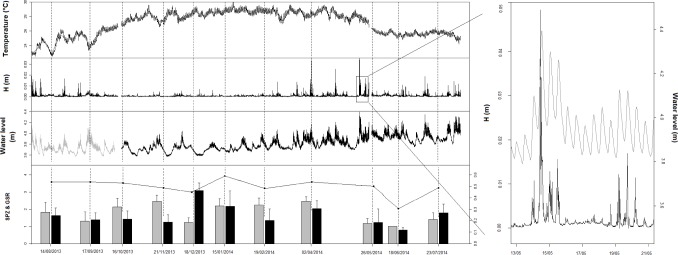
Physical characteristics of the Tatakoto lagoon from August 2013 to July 2014. Dashed vertical lines indicate when gonads were sampled. The lower panel displays values for the maturity indices SPZ (grey) and GSI (black), as well as the relative proportion of male and female tissues (GSR, black joined dots) obtained at each gonad sampling event. The water level line is greyed out before 15/10/2013 as the sensor was placed slightly deeper than usual on its pedestal (20 to 40 cm). Right panel is a zoom of water level (grey) and mean wave height (black) for the period 13–21 May.

Sexual maturity, as represented by GSI, at Tatakoto was found to be constant between August and November 2013, then more variable the following months. The index (mean ± SD) was estimated at 0.47 ± 0.07 in December 2013, declining to 0.12 ± 0.02 in June 2014 (Fig 5). This decline followed a period of increased water levels and concomitant decline in temperature (Fig 5). The index of male maturity (SPZ) was variable throughout the study period, with no spermatozoid found in the gonads of all 15 giant clams collected in June 2014, despite a sampling protocol geared toward small (i.e., male) individuals (i.e., low GSR). Despite the irregularity in sampling at Tubuai, similar GSI values were obtained in November 2013, September 2014, and December 2014, when temperatures are typically on the rise. In contrast, values were found to be more variable during the seasonal decline in temperature (see S1 Fig). In all instances, the male maturity index (SPZ) and the female maturity index (GSI) did not always show synchronic patterns.

### Natural mortality

**Fig 6:**
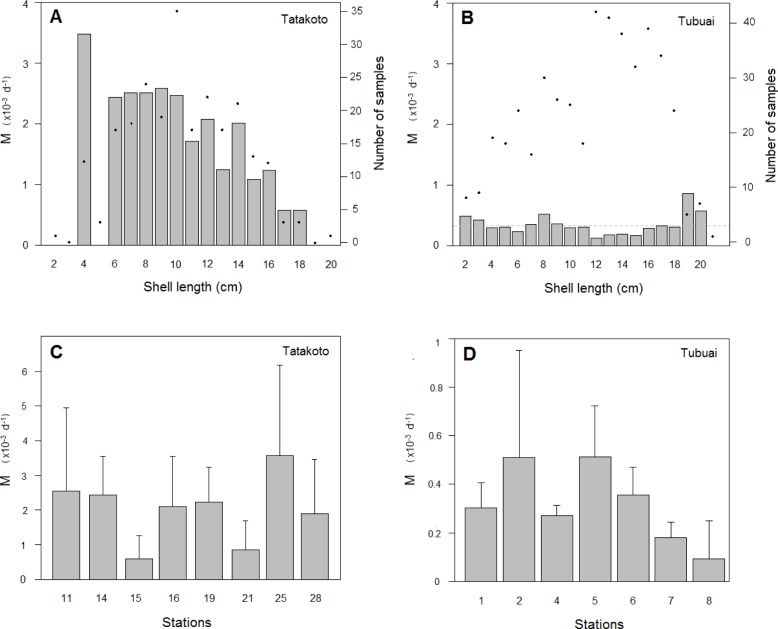
Instantaneous rate of natural mortality (*M*) for the two studied sites over the course of the study period (2012–2014). (A-B) Instantaneous rate of natural mortality (*M*) calculated per shell length class for Tatakoto (A) and Tubuai (B). Black dots display the number of samples used to calculate M. (C-D) Instantaneous rate of natural mortality (*M*) calculated for each station (mean ± SD over depth and survey time periods for Tatakoto (n = 4 for stations 11, 14 and 19; n = 8 for others) and over survey time periods for Tubuai (n = 3)).

At lagoon-scale, instantaneous rates of natural mortality (*M*) per shell length class and over the entire study period differed markedly between Tatakoto and Tubuai (Fig 6A and 6B). Despite a lower sampling effort applied to small and large individuals, *M* was relatively constant across all shell length classes at Tubuai (p = 0.819), whereas it decreased significantly with shell length at Tatakoto (p < 0.001). Natural mortality did not show any seasonal trend (warm season *versus* cold season) (p = 0.906 and p = 0.914 for Tatakoto and Tubuai respectively; see Table 3) or clear relationship with temperature, mean wave height or lagoon water level (see S2 and S3 Figs).

**Table 3:** ANOVA results for differences in instantaneous natural mortality rates (*M*) between sampling structuring factors.

Site	Factor	Df	F	p.value
Tatakoto	Station	7	3.797	<0.01**
	Season	1	0.014	0.906
	Depth	1	0.592	0.448
	Station × Season	7	1.726	0.142
	Season × Depth	1	0.600	0.445
	Station × Depth	4	1.192	0.335
Tubuai	Station	6	1.071	0.458
	Season	1	0.012	0.914
	Station × Season	6	0.458	0.819

** Denotes a significant difference with a type I (α) error below 0.01 (1%).

At intra-lagoon-scale, and for Tatakoto, the instantaneous rates of natural mortality (*M*) were significantly lower (p < 0.01) at station 15 and station 21 (*M* = 5.9×10^−4^ ± 6.7×10^−4^ and *M* = 8.5×10^−4^ ± 8.3×10^−4^ respectively; Table 3, Fig 6C) than for the remainder of tagging stations (*M* > 1.9×10^−3^). Both stations are located in close proximity to the narrow and shallow channel in the fringing reef connecting the lagoon with the ocean. At Tubuai, there were no significant differences between stations (p = 0.458), despite lower values in *M* for patch reefs in the eastern part of the lagoon (stations 7 and 8, Fig 6D).

## Discussion

In this study, we highlight notable differences in estimated growth rates and natural mortality for *T*. *maxima* between the lagoons of Tatakoto and Tubuai. These differences may be due to a number of biotic and abiotic factors, which we discuss below.

### Shell growth

At regional and lagoon-scales, we found greater shell growth rate during the warm season compared to the cold season at both sites. Previous studies have shown that bivalve growth rate and its variability increase with temperature [32], reaching erratic values when temperature is too high [33]. Based on available data, it is difficult to determine whether the lower growth rates recorded in the cold season were solely due to lower temperatures, or if other season-related processes might be at play (e.g., lower irradiance). Seasonality in shell growth was also observed for *Hippopus hippopus* in New-Caledonia [33] and for *T*. *maxima*, *T*. *derasa*, *T*. *squamosa*, *H*. *hippopus* and *T*. *gigas* in Australia [34, 35]. These results support our conclusions of greater growth rates for *T*. *maxima* during the warm season compared to the cold season in French Polynesia.

The shell growth rate of bivalves is often reported as a function of their shell length or age. For giant clams, the acquisition of energy may influence the relationship between shell growth rate and shell length since the number of zooxanthellae per unit of flesh decreases with age [36]. Our results showed the relationship between shell growth rate and shell length to be weak (R^2^ = 0.13 and 0.44 for Tatakoto and Tubuai respectively) because of high individual variability and measurement error. However, findings did suggest slower growth at Tatakoto compared to Tubuai and similar maximum sizes for the two lagoons (Fig 4A and 4B). These results are contrary to a previous study, which reported higher growth rates for small *T*. *maxima* in the Tuamotu atolls compared to the Austral islands (*k* = 0.135 and 0.145 for Tatakoto and Fangatau *versus* 0.068 for Tubuai) and lower theoretical maximum sizes (*L*_∞_ = 21.85 for Tatakoto and Fangatau against 24.7 for Tubuai) [18]. The differences in results may be due to the fact that small giant clams were under-represented in our sampling protocol.

Growth curves reported in the literature usually suggest that old individuals (> 10 years) are characterized by smaller shell length in atoll lagoons compared to other reef systems (Fig 4B). The growth curve generated from data collected at Tatakoto was similar to that observed in the Cook Islands (Aitutaki, Manihiki, and Suwarrow open atolls) [25]. Lower maximum sizes were also reported for the open Tongareva atoll (Cook Islands, 20.2 cm, [37]), for the semi-closed atolls of Reao, Pukarua, Napuka (20 cm, 22 cm, 23 cm respectively, [7]), and for the closed atoll of Takapoto (< 13 cm, [31]), compared to other reef systems (e.g., 30 cm at Tonga, [22]; 33 cm at Fiji, [38]; 31 cm at Ningaloo reef in Australia, [39]). The low sizes reported for bivalves in some atolls of the Tuamotu archipelago lead some authors to speak of “dwarfism of the macro-fauna” (e.g., Takapoto, [31]), and may be related to oxygen supply [40] or differences in food availability, if food (e.g., plankton and suspended organic matter) for example were to be found in lower concentrations in atoll lagoons than in open lagoons of Austral islands due to lower water renewal rates and larger populations of filter feeding bivalves [5]. At this stage however this remains a hypothesis warranting further research to be tested.

At intra-lagoon scale, higher growth rates and higher variability in growth were recorded at Tatakoto for giant clams located in shallow areas, corroborating other studies’ findings [41]. Considering the small differences in temperature observed between the sensors deployed at various depths (i.e., no strong thermocline), the differences in shell growth observed between depths are probably the result of different light intensities. The distribution of micro-habitat, in particular the density of branching corals, is a function of depth and may also influence giant clam shell growth (e.g., shadow effect). At Tubuai, despite a high variability observed at individual scale, lower mean growth rates were recorded on the reef flat and crest habitats in the south/southeast part of the lagoon that is more exposed to waves. Several studies have also highlighted the negative influence of hydrodynamics on giant clam shell growth [42]; this may be related to the decrease in the ability of benthic and heterotrophic marine species to catch suspended organic matter in strong currents [43].

### Reproduction

To date, very few studies have looked into the reproductive activity of giant clams [27, 44]. In particular, spawning periods and stimuli for spawning events in natural environments have not been clearly identified [45]. Some authors have suggested that the reproductive activity of giant clams in semi-closed atolls is linked to temperature changes brought about by periods of high and low lagoon water renewal (i.e., good and poor connections between the lagoon and the open ocean due to a combination of wind and swell regimes) [5, 46]. The same authors also propose that these patterns may explain differences in the reproductive activity of giant clams between the semi-closed atolls of the Tuamotu Archipelago and the open lagoons of the Austral Archipelago.

In this study, we found a decline in maturity indices in May and June 2014 at Tatakoto, indicative of a mass spawning event over that time period. This spawning event followed a decline in temperature, associated with high mean wave heights and a greater connection between the lagoon and the ocean (Fig 5). We cannot tell if the spawning stimulus was the abrupt drop in temperature itself or some change in other chemical or physical properties of the water (e.g., salinity, food, nutrient, [47, 48]), or if spawning occurred throughout the entire lagoon. To address this, future studies should collect monthly samples from several stations distributed throughout the lagoon at both Tatakoto and Tubuai. Despite these limitations, our results support the hypothesis that water renewal is a crucial driver of *T*. *maxima*’s reproductive activity in enclosed reef systems [5, 46].

### Natural mortality

Estimates of instantaneous rates of natural mortality were higher at Tatakoto than at Tubuai, particularly for the small shell length classes (Fig 6A and 6B). It is unlikely that the high mortality rates observed at Tatakoto were the result of the tagging method used because: (i) no significant differences in survival were observed between marked and unmarked giant clams during an experiment specifically designed to address this concern (see S1 Appendix in Supporting Information); (ii) the mortalities recorded in our study were not exclusive to the monitoring campaign that immediately followed tagging; (iii) Gilbert (2005) [18] also highlighted higher mortality rates in 2004/2005 at Tatakoto (from 29% to 85% in 315 days) and Fangatau (from 2.4% to 23% in 280 days) compared to Tubuai (from 3% to 7% in 311 days), especially for small individuals and despite a similar tagging method implemented at all sites; (iv) monitoring densities at lagoon-scale from 2012 to 2014 also suggests low mortality at Tubuai, but high mortality at Tatakoto (see S4 Fig). Thus, the higher mortality rates at Tatakoto than Tubuai observed in this study likely resulted from natural processes.

Factors that may cause elevated natural mortality rates among juvenile invertebrates include: disease, predation, competition, loss of energy, and unfavourable physical and chemical properties of seawater (e.g., desiccation, temperature, salinity, wave exposure, solar radiation) (see [49] for a review). Parasites and pathogens have induced the collapse of numerous populations of exploited bivalves [50; 51]). A population of parasites can grow from several individuals to thousands of individuals within several months in an aquaculture setting, where predators are absent [52]. Among the various parasites and pathogens known to affect giant clams (see [50] for an exhaustive list), ciliated protozoans are capable of inducing mass mortalities of juveniles (e.g., from 25% to 57% in several months for *T*. *gigas*; [53]). At Tatakoto, the parasite *Perkinsus sp*. was detected in 2012. This parasite is known to affect juveniles [54], but infected clams had lower parasite loading at Tatakoto compared to other lagoons in French Polynesia (e.g., Hao, Aratika). It is therefore unlikely to have caused the high mortality rates observed at Tatakoto.

The thin and delicate shells of small giant clams make them more vulnerable to predators [4]. Some authors have suggested the existence of a refuge size, usually around 10–15 cm, beyond which natural mortality induced by predation (excluding fishing pressure) decreases [55]. During a program of culture and release of giant clams in the Cook Islands, Waters et al. [56] monitored a pool of 1 year-old and 3 year-old giant clams. The authors reported survival rates ranging from 76 to 100% for 3 year-old individuals (mean shell length 50.2 ± 0.64 mm) and from 40 to 63% for 1 year-old individuals (14.4 ± 0.36) when giant clams were protected from predators with enclosures. By contrast, they reported survival rates of 40% (for 3 year-old) and 15% (for 1 year-old) when giant clams were exposed to predators. At Tatakoto, high abundances of octopus (*Octopus cyanea*) were regularly observed during field trips, as well as high predation rates on small giant clams by numerous fish (Labridae and Tetraodontidae) and gastropods (Pyramidellidae). The presence of predators and parasites in atoll lagoons may follow cyclical predator-prey relationships, with high abundances possibly the result of previously high giant clam densities [8]. However, this hypothesis remains unverified.

Intra-specific competition related to density-dependent processes can also negatively influence the natural mortality and shell growth of bivalves [57]. In the late 90s, early 2000s, density-dependent processes were identified as the main factor involved in the high mortality rates observed at Tatakoto and Fangatau [58]: many individuals were shaded by conspecifics due to the high densities registered and frequently had parts of their mantle bleached [5]. This was a realistic hypothesis because, at the time, densities were in the order of tens to hundreds of individuals per square metre. In 2012–2014, however, densities were an order of magnitude lower than during the previous decade, and it is much less likely that the high mortalities recorded in our study were a result of intra-specific competition. Alternatively, the higher mortality rates recorded at Tatakoto may be due to adverse benthic environmental conditions linked to the type of substrate. At Tatakoto, the latter consists essentially of loose, old giant clam shells. These can easily roll/move and may increase mortality rates of live animals. At Tubuai, lower survival rates were also observed on the exposed reef flat and crest (station 2, 5, and 6, Fig 1) compared to other stations, where waves frequently displaced entire coral blocks.

Finally, the higher mortality rates recorded at Tatakoto compared to those recorded at Tubuai may be the result of contrasting physical characteristics of lagoon waters between the two sites. In this study, we highlighted differences in temperature, wave exposure, tidal slope, and water level variability between both locations, partly due to their distinct reef aperture configuration (semi-closed atoll *versus* island). The reef pass and submerged reef flat at Tubuai allow for increased exchange between lagoon and oceanic waters. In this highly open lagoon, water levels are permanently driven by the tide (Fig 3). By contrast, Tatakoto’s atoll rim is closed in its northern part and segmented in its southern part by several shallow channels (*hoa*) that allow for oceanic and lagoon waters to mix during periods of high southerly swell (more common between March and September) and east/southeasterly winds. We found that a 10-day period of low waves inside the lagoon (i.e., low wind and low swell from the southeast) was associated with a decrease in lagoon water level and an increase in water temperature. At intra-lagoon scale, the lowest mortality rates were recorded at stations 15 and 21, located in close proximity to where the lagoon and ocean connect, while the highest mortality rates were recorded in areas subject to greater variations in temperature and more intense warming. This suggests that high water renewal rates and the associated physico-chemical water characteristics (e.g., low turbidity, high oxygen supply) in these areas increase survival. These results strengthen the idea that a prolonged period of “lagoon closure” (i.e., low water renewal) may decrease giant clam survival throughout the lagoon.

### Giant clam population dynamics and reef type

Our study suggests that reef aperture and lagoon water renewal play an fundamental role in giant clam life history, with significant differences recorded in shell growth rates, mortality rates and fertility rates between open *versus* enclosed atoll lagoons. While our study focused on two lagoons in French Polynesia, our work supports the hypothesis that key giant clam biological processes are related to reef type and lagoon configuration [3]. The work implemented here may bring new insights and promising perspectives to understanding the variability of giant clam life traits and population dynamics for many other lagoons throughout their geographical range in the Indo-Pacific.

## Supporting Information

S1 Appendix Complementary study on giant clam mortality at Tatakoto. (DOCX)

S1 Fig Monitoring of gonad maturity indices at Tubuai. A-2013: April 2013; N-2013: November 2013; S-2014: September 2014; D-2014: December 2014; A-2015: April 2015. SPZ (grey): male maturity index. GSR (red): Gonadal Sex-Ratio. GSI (black): Gonado-Somatic Index.
(EPS)

S2 Fig Instantaneous rate of mortality (M) and physical characteristics of the lagoon of Tatakoto from November 2012 to October 2014. Dashed vertical lines indicate field trip during which the survival of giant clams was checked. The lower panel display values for the instantaneous rate of mortality (M) obtained at the end of each period. The water level line is greyed out before 15/10/2013 as the sensor was placed slightly deeper than usual on its pedestal (20 to 40 cm).
(EPS)

S3 Fig Instantaneous rate of mortality (M) and physical characteristics of the lagoon of Tubuai from April 2013 to December 2014. Dashed vertical lines indicate field trip during which the survival of giant clams was checked. The lower panel display values for the instantaneous rate of mortality (M) obtained at the end of each period.
(EPS)

S4 Fig Monitoring of stocks from 2004 to 2014 at Tatakoto and Tubuai. (EPS)
