# Supplementary material for: Growth, Survival and Reproduction of the Giant Clam Tridacna maxima (Röding 1798, Bivalvia) in Two Contrasting Lagoons in French Polynesia
Source: PLoS One. 2017 Jan 24;12(1):e0170565. doi: 10.1371/journal.pone.0170565 (PMC5261826; doi:10.1371/journal.pone.0170565)
Supplement: S1 Appendix — (DOCX) [file pone.0170565.s001.docx]

## Supporting Information S1

## Complementary study on giant clam mortality at Tatakoto

**Purpose**

Because of high mortality and loss of giant clams at Tatakoto, we implemented a complementary study to fix if these mortalities were due to (i) the method used for marking individuals, (ii) high predation by macro-fauna, or (iii) movement of giant clams.

**Method**

44 marked giant clams and 84 unmarked giant clams were dispatched among three treatments (cf. Fig. A2.1.) from October 2014 to November 2015 at station 14 (n=3 replicats per treatment). Two treatments (A & B) prevented giant clams from (A) moving/rolling outside of the study zone using 3×3 cm wiring; and (B) from being predated by macro-fauna using a 0,5×0,5 cm wiring. A third treatment (C) did not protect giant clams and refers to the blank if this experience. Giant clam state (i.e., living, dead, lost) was checked monthly by a local villager (M. Kamake), and the wiring washed regularly to maintain good light conditions in all treatments.

The proportion of living giant clam between the start (October 2014) and end (November 2015) of the experiment is expressed as a function of variables « treatment » and « marking » using a linear model (function lm in R.3.1.0.), differences of survivorship between factors were tested using an analysis of variance (function anova in R.3.1.0.). The normality and non-differences of variances were checked using normal “QQ plots” and “S-L plots » (function plot.lm in R3.1.0.).

Then, a power analysis was performed using the software G*Power 3.1.9.2. to calculate the type II error (β) and the statistical power associated to the anova. Three values were considered as effect size, referring respectively to a low effect size (0.10), medium effect size (0.25) and large effect size (0.40) according to the convention established by Cohen (see Faul et al. 2007)


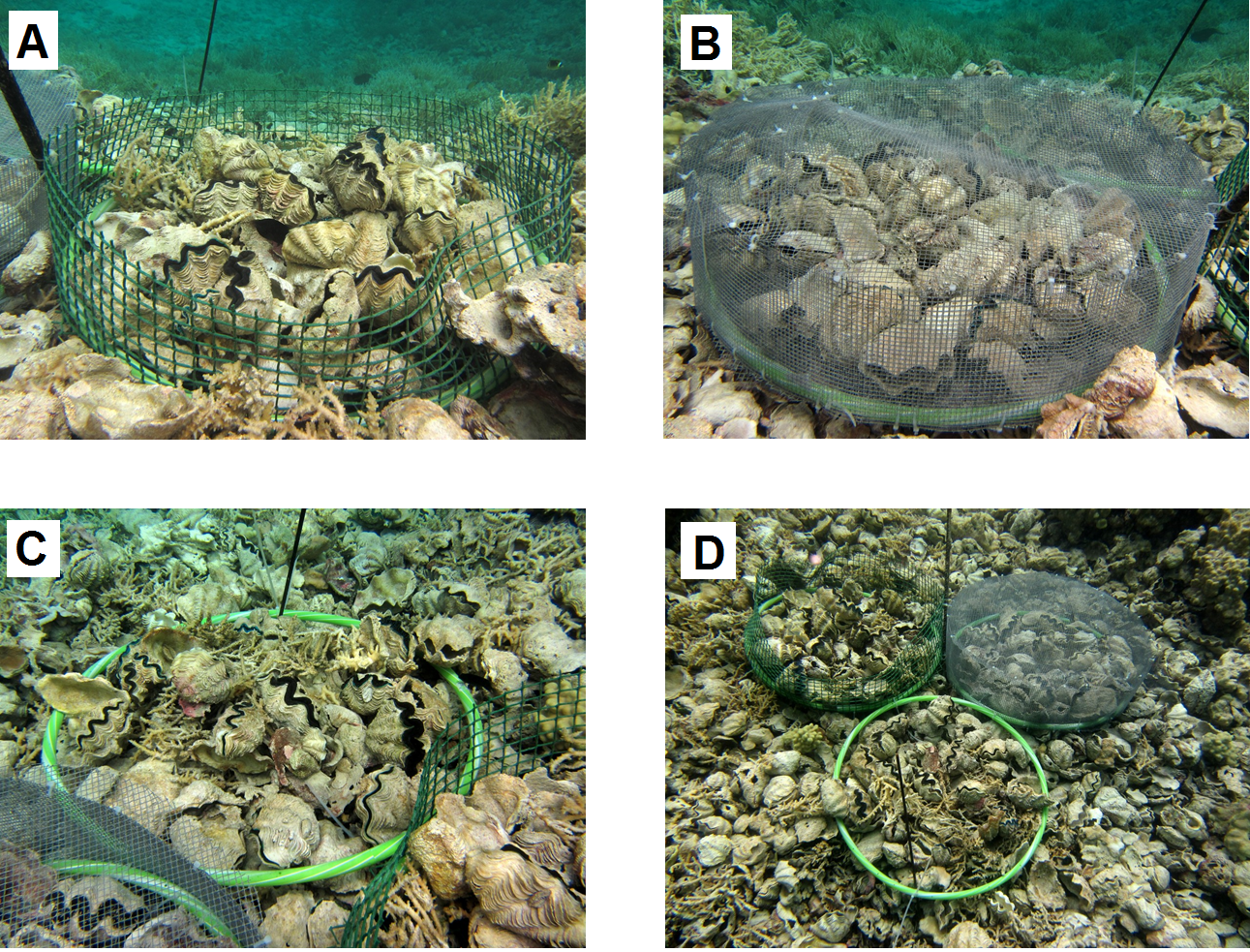


Figure A2.1. : Pictures of the three treatments applied to giant clams in this complementary study. A : Treatment (A) which isolate giant clams from moving/rolling out of the study area ; B : Treatment (B) which isolate giant clams from predators; C : Treatment (C) which do not provide any protection. D: overall view of one monitoring station. The study is based on three stations.

**Results**

No effect of treatment (F = 0,29 ; p = 0,76), nor marking (F = 1,34 ; p = 0,28), nor the interaction between treatment and marking (F = 0,99 ; p = 0,41) could be evidenced in this study (Fig. A2.2., Tableau A2.1.). Statistical power associated to the tests were low for small effect sizes (ES) (i.e., Power below 0,63), but sufficient for strong effect sizes (Power between 0.72 to 0.95) (Tableau A2.1.).


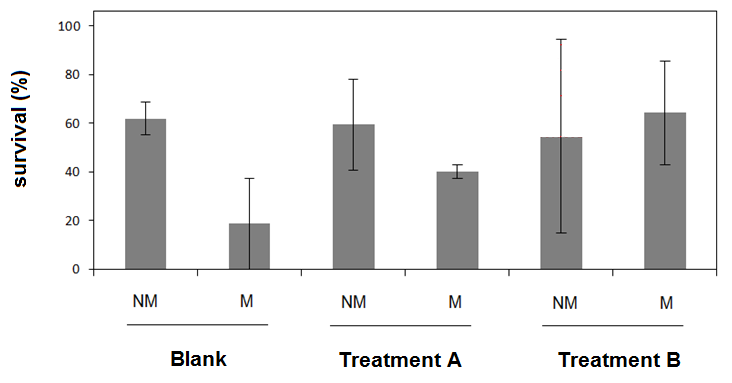


Figure A2.2. : Proportion of living giant clams after 13 month of monitoring (Octobre 2014-Novembre 2015) for the various treatments implemented at Tatakoto. Error bars are standard deviation (n=3).

Table A2.1. : Result of ANOVA which tests for differences of survival between treatments (A, B and C), marking effect, and the interaction between treatment and marking effect. (1-β) refers to statistical power associated to each test and is calculated using G*Power 3.1.9.2. ES : « effect size ».

| Factor tested | F | p.value | (1-β) | | |
| --- | --- | --- | --- | --- | --- |
|  |  |  | Small ES | Medium ES | Strong ES |
| Marking | 1,337 | 0,277 | 0,14 | 0,63 | 0,95 |
| Treatment | 0,289 | 0,756 | 0,12 | 0,52 | 0,91 |
| Marking×Treatment | 0,995 | 0,407 | 0,09 | 0,36 | 0,80 |
|  |  |  |  |  |  |

**Discussion**

The aim of this study was to explain the cause of high mortality (i.e., strong effect size) observed at Tatakoto. In this context the statistical power of our study is acceptable.

It is unlikely that the high mortality observed at Tatakoto were the result of the tagging method used because:

(1) no significant differences in survival were observed between marked and unmarked giant clams during this experiment specifically designed to address this concern.

(2) the mortalities recorded in our study were not exclusive to the monitoring campaign immediately following the tagging session.

(3) Gilbert (2005) also highlighted higher mortality rates in 2004/2005 at Tatakoto (from 29% to 85% in 315 days) and Fangatau (from 2.4% to 23% in 280 days) compared to Tubuai (from 3% to 7% in 311 days), especially for small individuals and despite a similar tagging method implemented at all sites. Therefore, we conclude that the high mortality rates observed at Tatakoto were the result of natural processes.

**References**

Faul F, Erdfelder E, Lang AG, Buchner A (2007) G*Power 3: A flexible statistical power analysis program for the social, behavioural, and biomedical sciences. Behavior research methods 39: 175-191.

Gilbert A (2005) Vers une gestion durable des bénitiers de trois lagons de Polynésie française : Fangatau, Tatakoto (Tuamotu est) et Tubuai (Australes). Rapport final. Institut de Recherche pour le Développement, Arue, French Polynesia.
